# Immune Escape Mechanism and Vaccine Research Progress of African Swine Fever Virus

**DOI:** 10.3390/vaccines10030344

**Published:** 2022-02-22

**Authors:** Zhaoyang Wang, Qiangyun Ai, Shenglin Huang, Yating Ou, Yinze Gao, Tiezhu Tong, Huiying Fan

**Affiliations:** 1 College of Veterinary Medicine, South China Agricultural University, Guangzhou 510642, China; zhaoyangwang@stu.scau.edu.cn (Z.W.); aqy@stu.scau.edu.cn (Q.A.); hsl@stu.scau.edu.cn (S.H.); ouyating@stu.scau.edu.cn (Y.O.); gaoyinze@stu.scau.edu.cn (Y.G.); 2 National and Regional Joint Engineering Laboratory for Medicament of Zoonosis Prevention and Control, Guangzhou 510642, China; 3 Key Laboratory of Animal Vaccine Development, Ministry of Agriculture, Guangzhou 510642, China; 4 Key Laboratory of Zoonosis Prevention and Control of Guangdong Province, Guangzhou 510642, China; 5 Guangzhou Customs Technology Center, Guangzhou 510623, China

**Keywords:** African swine fever virus, immune escape, immune response, vaccines

## Abstract

African swine fever virus (ASFV) is the causative agent of the epidemic of African swine fever (ASF), with virulent strains having a mortality rate of up to 100% and presenting devastating impacts on animal farming. Since ASF was first reported in China in 2018, ASFV still exists and poses a potential threat to the current pig industry. Low-virulence and genotype I strains of ASFV have been reported in China, and the prevention and control of ASF is more complicated. Insufficient understanding of the interaction of ASFV with the host immune system hinders vaccine development. Physical barriers, nonspecific immune response and acquired immunity are the three barriers of the host against infection. To escape the innate immune response, ASFV invades monocytes/macrophages and dendritic cells, thereby inhibiting IFN expression, regulating cytokine expression and the body’s inflammatory response process. Meanwhile, in order to evade the adaptive immune response, ASFV inhibits antigen presentation, induces the production of non-neutralizing antibodies, and inhibits apoptosis. Recently, significant advances have been achieved in vaccine development around the world. Live attenuated vaccines (LAVs) based on artificially deleting specific virulence genes can achieve 100% homologous protection and partial heterologous protection. The key of subunit vaccines is identifying the combination of antigens that can effectively provide protection and selecting carriers that can effectively deliver the antigens. In this review, we introduce the epidemic trend of ASF and the impact on the pig industry, analyze the interaction mechanism between ASFV and the body’s immune system, and compare the current status of potential vaccines in order to provide a reference for the development of effective ASF vaccines.

## 1. Introduction

In 1921, ASFV was first reported in Kenya, which caused about an 100% mortality in infected domestic pigs [1]. Subsequently, ASFV spread to most African countries south of the Sahara [2]. In 1957 and 1960, the African swine fever epidemic broke out in Portugal which was the first time to demonstrate that ASFV spread across continents and spread to other European countries, the Caribbean, and Brazil [3]. Except in Sardinia, the genotype I ASFV was eradicated in the 1990’s [4]. However, the genotype II ASFV was introduced to Georgia in the Caucasus region and started a new round of transmission in 2007 [5]. Afterwards, it spread to the Russian Federation, Ukraine and Belarus, and in 2014 it spread to Eastern European countries [4,5]. In 2018, the epidemic spread to Belgium, Hungary, Czech Republic, Romania, Bulgaria, Slovakia, and Serbia, as well as China and other parts of Asia (Mongolia, Korea, Vietnam, Laos, Cambodia, Myanmar, the Philippines, Hong Kong, and Indonesia) [3]. ASF outbreaks are still trending regionally, with new regional reports of ASF outbreaks. In September 2019, Timor-Leste reported the first outbreak of African swine fever in Oceania, followed by Papua New Guinea (March 2020) [6,7]. In July 2021, ASF reappeared in the Americas, first in the Dominican Republic and later in Haiti [8,9]. In January 2022, ASF genotype II was notified in mainland Italy [9]. In January 2022, two new countries also reported their first cases of the disease: North Macedonia in Europe and Thailand in Asia [9]. According to OIE, from January 2020 to January 2022, ASF outbreaks were reported in 35 countries or regions around the world, including 4767 cases (1043334 animals lost) in domestic pigs and 18,262 cases (29970 animals lost) in wild boars [9]. It is worth noting that wild boar cases in Europe accounted for the vast majority (83.3%, 16743/20107), while wild boar cases in Asia accounted for a lower proportion of 59.4% (1519/2559) [9]. The wild boar reservoir of ASFV will present challenges to the eradication of ASF by vaccination programs.

In August 2018, the first ASF case in China was identified in Shenyang, Liaoning Province [10]. Since most pig farms in China are small-scale pig farms with very low biosecurity levels, the rapid spread of ASFV across the country has led to a serious reduction in the number of live pigs and caused heavy losses to the pig industry [11]. The decline in the number of live pigs has led to a rapid rise in the price of pork in a short period of time, affecting the normal life of residents in China. After the outbreak of ASF, with the joint efforts of Chinese government leaders and industry practitioners, various pig farms tried to resume production. By improving the level of biosecurity, including optimizing the location of pig farms, strict disinfection measures, and strengthening ASFV testing on pig farms, ASFV-positive pigs were culled at designated locations, and the source of the infection was eliminated. Most pig farms finally successfully resumed breeding [12]. However, in order to improve the level of biosecurity, the measures taken by pig farms have objectively increased the cost of breeding. Therefore, there is an urgent need for vaccines for the effective control and eradication of ASF.

**Figure 1:**
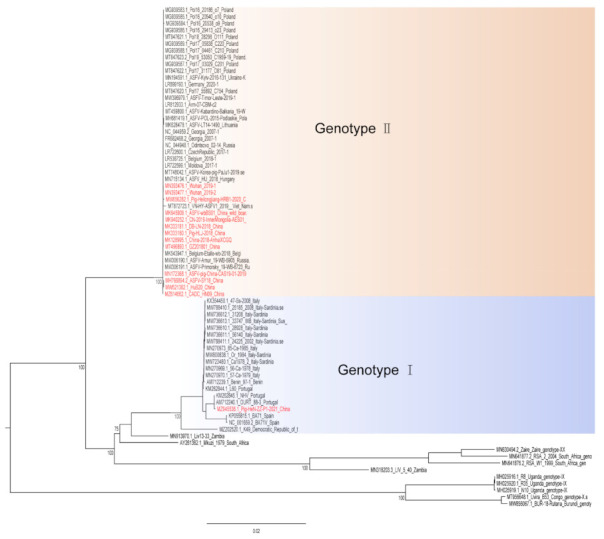
A phylogenetic tree was constructed based on the whole genome sequences of 74 strains in the GenBank database, after which datasets for the sequences were aligned using MAFFT (version 7.149) program [32]. Maximum likelihood (ML) phylogenies for the codon alignment of the genome sequences were estimated using the GTRGAMMA nucleotide substitution model in the IQ-TREE 1.68 software [33]. Node support was determined by nonparametric bootstrapping with 1000 replicates, and the phylogenetic tree was visualized in the Figtree (version 1.4.3) program (http://tree.bio.ed.ac.uk/software/Figtree/) (accessed on 18 January 2022). Types written in red indicate Chinese isolates.

Hemadsorption (HAD) refers to the ability of ASFV-infected macrophages to adsorb erythrocytes, and non-hemadsorption (non-HAD) refers to the inability of ASFV-infected macrophages to adsorb erythrocytes. ASFV has spread for more than three years in China, and its epidemic trend has become more complicated with the emergence of non-hemadsorbing (non-HAD) naturally attenuated strains of ASFV [13,14,15]. The initial clinical symptoms of domestic pigs infected with low-viral strains are not obvious, which is difficult to diagnose [13,14]. Comparative analysis showed that the genome of the low-virulence strains had mutations, deletions, insertions or substitutions of short fragments, including the deletion or mutation of the CD2v (EP402R) gene, which is related to the HAD phenotype [16]. In addition, genotype I strains have also been reported in China (Figure 1) [14]. The potential for endemic ASF outbreaks is increasing due to the widespread distribution of wild boars in China. Researchers are actively conducting ASF vaccine-related research, including the evaluation of live attenuated vaccines (LAVs) vaccines by knocking out virulence genes, and the screening of antigens with protective effects to develop subunit vaccines, vector vaccines, etc. [17,18,19,20,21,22,23,24,25,26,27,28,29,30]. ASFV has evolved the ability to manipulate host immune responses by encoding many immune escape genes, including regulation of IFN expression, inhibition of autophagy, and apoptosis. In addition, ASFV affects the antigen presentation and activation of lymphocytes by invading monocytes-macrophages and DCs, thereby affecting the immune system of the entire body [31]. This review summarizes the escape mechanism of ASFV from the immune response and the progress of ASF vaccines, hoping to provide help for future ASF vaccines research.

## 2. The Major Defense Mechanisms and ASFV Interactions

### 2.1. Physical Barriers

The skin barrier is the most effective physical defense of the host to block entry of pathogens. The physical barriers of the body’s defenses include intact skin, surfaces of the respiratory and gastrointestinal tracts and include processes of self-cleaning (coughing, sneezing and mucus flow in the respiratory tract, vomiting, diarrhea, and urination of the urinary system, etc.), and normal flora on the skin surface and in the gut. The transmission of ASFV among pigs is mainly direct contact transmission, including transmission through mouth-nose contact and short-distance aerosol transmission [34]. In addition, the presence of soft tick bites in pig farms can also cause the transmission of ASFV [35]. Therefore, it is necessary to improve animal welfare in the farm, and increase the resistance of animals by reducing the breeding density. Pig farms should strengthen environmental sanitation and disinfection to block the spread of the virus. On the contrary, for infected animals, the self-cleaning of the body is also conducive to virus spread. It is necessary to regularly collect and detect the presence of ASFV in pig herds, and to ensure the removal and safe destruction of positive pig herds to eliminate the source of infection [12].

### 2.2. Innate Immunity

Sentinel cells, such as macrophages, dendritic cells, and mast cells, elicit inflammatory responses by recognizing pathogen-associated molecular patterns (PAMPs) of microorganisms through pattern recognition receptors. The inflammatory response involves the activation and directed migration of various cells from the blood to the site of invasion, especially neutrophils and macrophages. The pattern recognition receptors that recognize ASFV mainly include TLR3 that recognizes viral dsRNA and cGAS-STING that recognizes viral DNA in the cytoplasm [36,37,38]. When TLR3 binds to dsRNA, it transmits signals to cells, resulting in increased expression of nuclear factor kappa-B (NF-κB), which in turn activates IL-1, IL-6 and TNF-α and other cytokine expression [36]. The cGAS-STING system recognizes virus DNA, and the virus-infected (mainly plasmacytoid dendritic cells) cells produce IFN-I [38]. IFN-I acts on virus-infected cells to inhibit virus growth.

**Figure 2:**
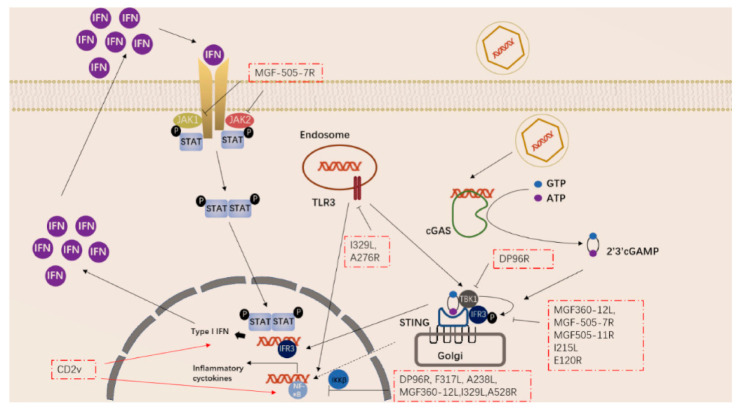
ASFV genes regulate innate immune signaling pathway.

ASFV has also acquired many genes in evolution that can regulate the inflammatory response in the early stage of the body. ASFV-encoded genes also primarily inhibit the actions of TLR3 and the cGAS-STING system (Figure 2). Studies have shown that the I329L and A276R proteins encoded by ASFV have immunosuppressive effects [39,40]. I329L (the homologous protein of TLR3) interacts with the adaptor protein TRIF to inhibit the activation of NF-κB and IRF3, while A276R can also inhibit the recognition of TLR3 [37,39]. In addition, another study showed that TLR expression was decreased after ASFV infection of macrophages [40].

ASFV encodes multiple genes involved in suppressing the cGAS-STING signaling pathway. In 2013, Correia et al. reported that A528R (MGF530) could inhibit the expression of IFN-β by targeting IRF3 and NF-κB or through the JAK-STAT pathway [39,41]. In 2018, Wang et al. found that DP96R targets TBK1 and IκB kinase beta (IKKβ) to negatively regulate IFN-I expression and induction of NF-κB signaling [42]. In 2020, Zhuo et al. demonstrated that MGF360-12L can block the interaction between p65 and KPNA2, KPNA3, and KPNA4, and can interfere with the nuclear translocation of NF-κB [43]. Five immunosuppressive genes were reported in 2021, including E120R, F317L, MGF505-7R, MGF505-11R, and I215L [44,45,46,47,48,49]. E120R can interact and block the activation of IRF3, thereby inhibiting the expression of IFN-β [44]. F317L inhibits IKKβ phosphorylation, thereby reducing the phosphorylation and ubiquitination of IkBα, thereby inhibiting the activation of the NF-κB pathway [45]. Li et al. reported that MGF-505-7R promoted the expression of autophagy-related protein ULK1 to degrade STING, and inhibited IFN-γ-mediated JAK1 and JAK2-mediated signaling pathways [46,48]. Similarly, Yang et al. reported that MGF505-11R interacts with STING, degrades STING expression through lysosome, ubiquitin-proteasome and autophagy pathways, and can inhibit the phosphorylation of TBK1 and IRF3 stimulated by cGAS/STING overexpression [47]. pI215L recruits RNF138 to inhibit K63-related TBK1 ubiquitination, which has an inhibitory effect on IFN-I production [49]. It is worth noting that CD2V can activate NF-κB and induce activation of IFN signaling pathways in porcine lymphocytes/macrophages [50]. These immunosuppressive genes are potential target genes for the preparation of live attenuated vaccines.

### 2.3. Acquired Immunity

#### 2.3.1. Antigen Processing

Dendritic cells and macrophages are both sentinel cells of innate immunity and efficient antigen processing cells. DCs are the only antigen-presenting cells capable of activating T cells that have never been exposed to an antigen (naive T cells). ASFV invasion of DCs may seriously affect their antigen processing and delivery, resulting in immunosuppressive responses [51]. Antigen processing breaks down antigens into small polypeptides, which are subsequently attached to professional antigen receptor-major histocompatibility complex molecules (MHCs).

Exogenous antigen processing begins with phagocytosis of the antigen to form endosomes, which are gradually acidified during intracellular migration and maturation, followed by fusion with protease-containing lysosomes [52,53]. The ingested peptides are degraded into fragments of different lengths by proteases, and some peptide fragments are combined with MHC-II molecules and presented to CD4^+^ T cells. The invasion process of ASFV utilizes the exogenous processing pathway of the cell. After acidification of the endosome, ASFV sheds its capsid, fuses its inner capsule with the restrictive endosomal membrane, releases its core capsid into the cytoplasm, and then releases its DNA [54].

Ubiquitin molecules bind to lysine of endogenous abnormal proteins. Ubiquitin chains are recognized by the proteasome and cleaved into fragments of 8 and 15 amino acids in length. In addition, the activity of the proteasome is regulated by cytokines such as IFN-γ and caspases. In fact, ubiquitination modifications have been used in ASFV vaccine research. A DNA vaccine fused to the extracellular domain (sHA) of ASFV p30, p54, and CD2v induced potent humoral and specific T cell responses in pigs, but was not protective [55]. In addition, ubiquitination of the above three genes may provide partial protection by improving the presentation of major histocompatibility complex (MHC) class I antigens [55]. This property of ubiquitination could be applied to the design of future ASF vaccines.

#### 2.3.2. Antigen Presentation

The antigen presentation process includes endogenous presentation pathway (MHC-I pathway), exogenous presentation pathway (MHC-II pathway) and cross antigen presentation pathway. MHC-I is present in all nucleated cells and presents endogenous antigens to CD8^+^ T cells. MHC-II exists only in professional antigen-presenting cells, dendritic cells, macrophages and B cells, and its function is to present exogenous antigens to CD4^+^ T cells.

Exogenous molecules elicit adaptive immunity by binding to the antigen-binding groove of at least one MHC molecule. MHC-class Ia and class II molecules are polymorphic, and one MHC molecule is estimated to bind to about 2500 different polypeptides. Therefore, MHC alleles can determine the susceptibility of the body to infectious diseases based on the adaptive immune response to the infecting pathogen. Screening for specific MHC haplotypes can be used to develop ASFV-resistant domestic swine lines. In March 2020, a Chinese research team reported that ASFV-resistant domestic pigs LS-2 were significantly resistant to oral infection with the 10^6.0^ TCID50 ASFV SY18 strain. There were significant improvements in the rate of infection, viremia, clinical symptoms, and antibody response after challenge, and there were also significant differences in the expression of inflammatory factors, which is of great significance for the prevention and control of ASFV [56]. However, MHC polymorphism is an evolutionary conserved feature making it difficult for pathogens to evade from immune recognition; if one resistant MHC haplotype is clonally expanded, this may bear risks for the other epizootic pathogens to come.

EP153R of ASFV can affect the trafficking of MHC-I from the endoplasmic reticulum to the cell membrane [51]. In addition, ASFV infection of cells resulted in increased expression of SLA-DOA and SLA-DOB, which inhibited the binding of antigenic peptides to MHC-II [40]. However, ASFV can inhibit the expression of MHC-I, NK cells will not receive inhibitory signals, and cells infected with ASFV may be killed [57]. Similar to T cells, NK cells induce apoptosis by activating intracellular caspases cascades through both exogenous and endogenous pathways. Interestingly, Franzoni et al. showed that attenuated or avirulent strains, but not virulent strains, were able to downregulate the expression of MHC-I molecules on MoDCs [58]. Similar results were observed in experiments infected with un-activated (moMΦ) and alternatively (moM2) activated monocyte-derived macrophages [59]. These studies of virulence ASFV have evolved mechanisms for covert replication, which may escape the killing effect of NK cells [58,59].

#### 2.3.3. Antibody Induction

Activation of B cells requires the stimulation of multiple signals. Antigen binding to the BCR is a necessary first step in triggering a B cell response, but is often insufficient to trigger antibody formation. Activation of B cells requires co-stimulation of helper T cells and cytokines. However, for helper T cells to function, they need to first present antigens to the helper T cells before they can provide costimulatory signals. The antigen can be captured and processed by DCs, macrophages and B cells and presented to T cells. The target cells of ASFV are mainly macrophages and DCs, and their antigen-presenting ability may be reduced after a virus infection [60,61,62]. The body may activate B cells through other pathways. After capturing the antigen, B cells process the antigen and present it to helper T cells. The antigen-presenting ability of B cells is much smaller in comparison with DC cells.

Responding B cells can differentiate into plasma cells, which secrete large amounts of antibodies. The main functions of antibodies include blocking the adsorption of viruses to target cells, stimulating the phagocytosis of macrophages, and stimulating complement-mediated viral decomposition. However, antibodies elicited by ASFV infection cannot completely neutralize ASFV, and about 4–13% of the virus cannot be neutralized [63,64]. An experiment revealed that the incomplete neutralization of ASFV may be caused by virus aggregation [64]. In addition, non-neutralizing antibodies in the ASFV infection may result in Antibody-Dependent Enhancement (ADE) [65]. Therefore, ASFV vaccines need to eliminate those antigens or epitopes that induce non-neutralizing antibodies.

#### 2.3.4. Cellular Immune

Cell-mediated immunity clears abnormal cells and intracellular pathogens. Endogenous antigens are first cleaved into small polypeptides and then inserted into the antigen-binding groove of the major histocompatibility complex (MHC) class I. When presented to the cell surface, the antigen binds to the antigen receptor of T cells. After CD8^+^ T cells are fully activated, they leave the lymphoid organs and search for infected cells by themselves. When activated CD8^+^ T cells recognize MHC-antigen complexes expressed on another cell, and cytotoxic T cells force the apoptosis of virus-infected target cells. In addition, NK cells fight against viral infection by directly killing virus-infected cells, inducing antibody-dependent cell-mediated cytotoxicity (ADCC), or secreting cytokines (such as IFN and TNF-α). A study has shown that asymptomatic pig herds infected with NH/P68 strain significantly enhanced NK cell cytotoxicity and resisted virulent ASFV Lisbon 60 challenge [66]. The genes currently known to inhibit apoptosis are A224L, EP153R, A179L, and DP71L, mainly by inhibiting the death receptor pathway, the mitochondrial apoptotic pathway and protein kinase RNA-like ER kinase (PERK) pathways (endoplasmic reticulum stress response) [67,68,69,70]. Granzyme B is one of the main effector molecules, and can directly act on caspase-3 to induce apoptosis. ASFV-encoded EP153R and A224L can inhibit the activity of caspase-3 [67,68]. However, granzyme B can trigger apoptosis through ICAD/CAD [71]. In addition, it has been reported that ASFV can inhibit the expression of cytotoxic T cells perforin at 5 dpi [72]. ASFV may partially inhibit perforin/granzyme-induced apoptosis. Furthermore, when the host cell senses abnormal signals, eIF2 is phosphorylated, which shuts down the protein synthesis system. DP71L of ASFV promotes the replication of the virus in cells by recruiting PP1 to dephosphorylate eIF2 [70]. When ASFV replication is completed, the mitochondrial apoptosis pathway is activated through the structural protein p54 encoded by E183L, which promotes cell lysis to start a new round of infection and replication [73]. ASFV encodes related proteins to regulate apoptosis and promote the replication, proliferation and spread of ASFV, which is also one of the strategies to achieve immune escape.

In conclusion, effective ASF vaccines can induce the production of neutralizing antibodies that block the entry of ASFV into target cells or induce ADCC. Therefore, screening for target epitopes of neutralizing antibodies is required. In addition, vaccination induces the body to produce Cytotoxic T lymphocyte cells (CTLs) that can specifically recognize abnormal cells. Similarly, it is also necessary to screen for antigenic epitopes that can induce specific cellular immunity. In fact, this is also one of the current research directions [74,75,76,77,78]. However, whether the effective combination of antigenic epitopes is effective still needs much experimental verification.

## 3. ASF Vaccines

### 3.1. Live Attenuated Vaccines (LAVs)

LAVs can generally be divided into natural attenuated strains and artificial gene deletion strains, and the latter is made by deleting certain virulence genes. Compared with other types of ASF vaccines, LAVs can provide complete homologous and partial heterologous protection [79]. The most promising LAVs vaccine candidates are summarized in Table 1. Chen et al. reported that a seven-gene deletion LAV had a good protective effect [17]. In addition, Zhang et al. reported that deleting L7L-L11L attenuates ASFV. In vaccination experiments, these attenuated strains conferred 100% protection against homologous challenge [18]. LAVs have experienced a trend from multiple gene deletions to single gene deletions. Protection induced by immunization with LAVs correlates with the level of replication of the LAVs in addition to the number of immunogenic genes expressed. If the replication of LAVs is severely attenuated, then the LAVs will not be immunogenic. LAVs with single gene deletions express more genes and may cause better protection. ASFV-G-∆I177L could achieve 100% protection through oral and injection routes, and field trials have been completed in Vietnam [19,20,21]. Similarly, ASFV-G-ΔA137R and SY18ΔI226R were proven to confer 100% protection [22,23].

Historically, attenuated strains were once used in Spain and Portugal, but widely caused chronic ASF infections in vaccinated pigs. Therefore, adequate experiments should be conducted to verify the effectiveness and safety of LAVs before extensive promotion, for example, future ASF vaccines should be distinguish between infected animals and vaccinated animals (DIVA) [80]. Ramirez-Medina et al. constructed a deletion strain of E184L to distinguish between infected animals and vaccinated animals (DIVA), but the deletion strain cannot provide complete protection [80]. LAVs are still far from commercialization due to the safety issue. LAVs may have the risk of virulence reversion during large-scale vaccination. Therefore, adequate experiments should be conducted to verify the effectiveness and safety of LAVs before extensive promotion.

In addition, the lack of cell lines for large-scale LAVs production is another difficulty. ASFV subculture can adapt to cell lines such as 293 and Vero, but the virulence and antigenicity of the adapted strains are weakened, and adapted strains cannot provide effective protection after immunizing pigs [81,82]. The adapted cells cultured in LAVs are primary cells, mainly including porcine alveolar macrophages (PAM) and porcine bone marrow cells (PBMS). Primary cells are not sufficient for large-scale production of LAVs. Borca et al. reported that ASFV-G-ΔI177L/ΔLVR could replicate efficiently in stable porcine cell lines with potent protection [83]. In addition, Takenouchi et al. reported a novel immortalized porcine macrophage cell line, IPKM, which was generated by transduction of primary PKM with lentiviral vectors encoding SV40LT and pTERT [84]. However, it is necessary to carefully test it before using IPKM as a production cell line for a live-attenuated vaccine strain.

Finally, the cross-protective ability of LAVs needs to be further evaluated. ASFV has mutations, duplications, and loss of certain sequences in the genomes of different genotypes or even different strains of the same genotype, which may lead to changes in virulence [85].

### 3.2. Subunit Vaccines

Subunit vaccines are generally in the laboratory research stage, and there is still a huge gap between practical applications. It has been confirmed that some ASFV antigens have a certain protective effect [24,26]. Antibodies against p72 and p54 can block the adsorption of ASFV, and antibodies against p30 can block the internalization of the virus [87]. The key to subunit vaccines is to screen for those antigens or those epitopes of antigens that have definite protective effects [88]. In the presence of non-neutralizing antibodies, the virus-antibody complex formed is infectious. The virus bound by the non-neutralizing antibody is phagocytosed by macrophages, and the virus can still grow in the macrophage at this time, and the virus bound by the non-neutralizing antibody accelerates the replication of the virus [89,90].

Previous studies have noted a critical role for CD8^+^ T cells in protection, as demonstrated by using the virulent Portuguese ASFV isolate OUR/T88/1 [91]. DNA vaccines as well as vector vaccines have been attempted as alternative ASF vaccine platforms. In theory, DNA vaccines as well as vectors have better immunogenicity because the antigen can be expressed intracellularly and via MHC I, which is important for CD8^+^ T cell activation. Neither DNA vaccine, vector vaccine, nor prime-boost immunization strategies can provide complete protection [56,92,93,94]. Luckily, Goatley et al. cloned B602L, p72, p30, p54, E199L, EP153R, F317L, and MGF505-5R into adenovirus vectors for primary immunization, and into MVA vectors for booster immunization, then found the protective function up to 100% in pigs [30]. However, low levels of infectious virus remained in the immunized pigs until the end of the trial [30]. Future experiments should determine whether pigs can clear the infection and whether these vaccinated animals shed infectious virus [30].

Similar to screening for antigens for neutralizing antibodies, screening is also required to identify antigenic epitopes capable of inducing ASFV-specific T cells. Bosch-Camós used in silico prediction of ASFV protein CD8^+^ T cell epitopes and predicted a 19-mer peptide from MGF100-1L [76]. Netherton et al. predicted peptides corresponding to 133 proteins encoded by OUR T88/3, and screened 18 viral proteins that could be recognized by lymphocytes of ASF-immunized pigs [78]. The specific antigenic epitopes are determined by software analysis and verification in combination with relevant experiments, which guides the selection of antigens in the development of ASF vaccines.

Recently, some research teams have conducted large-scale screening of subunit vaccine protective antigens and identified a group of antigens that can effectively block virus replication in pigs and protect cohabiting pigs from infection [95,96]. Part of the data showed that CD2V protein and p72 protein containing trimers make subunit vaccine compositions that can provide complete and safe protection to pigs [95].

### 3.3. Other Types of Vaccines

Inactivated vaccine is a safe and low-cost vaccine that is used in the prevention of many diseases [97,98,99]. However, the ASF inactivated vaccine does not provide protection [100,101]. Co-immunization of inactivated vaccines and adjuvants that stimulate cellular immunity also failed to provide protection [102].

The ASF outbreaks from 2016 to 2020 are dominated by wild boar populations in Europe, and wild boar infections have also been reported in Asia [103]. ASFV spreads in wild boar herds and may spread to domestic pigs. The use of oral vaccines in ASFV endemic areas may be a feasible measure to prevent ASFV in wild boars. Previous studies have demonstrated that oral live-attenuated vaccines could achieve effective protection in in the laboratory stage [20,104].

Critical progress in mRNA vaccines technology has occurred over the past two decades [105,106], which have recently presented distinguished effectiveness against COVID-19 [107,108]. mRNA vaccines are divided into non-replicating and self-amplifying (Alpha virus replicons). The mRNA expression of exogenous protein in the cytoplasm does not need to enter the nucleus, so it can be expressed in non-dividing cells or slowly dividing cells (such as dendritic cells). Compared with DNA vaccine, mRNA vaccines have higher safety and stronger immunogenic response in humans and large animals [109,110,111]. And for viral vector vaccines, mRNA vaccines have superior safety and applicability, and will not affect the effect of boosting immunity [112,113]. However, mRNA vaccines are also expensive to manufacture and transport, and there may be potential unknown risks. Compared to mRNA vaccines, self-amplifying RNA-replicons represent a cost-effective alternative that could become an interesting approach also in veterinary medicine.

## 4. Conclusions

ASFV has a complex five-layer structure and encodes 150 to 200 proteins, including many unknown functional proteins. Low-virulence and genotype I strains of ASFV have been reported in China, and the prevention and control of ASF is more complicated. The major defense mechanisms of animals have three lines of defense against the invasion of microorganisms. The first line of defense is a simple physical barrier that can resist most pathogens. The second line of defense is the non-specific immune response, which mainly includes neutrophils, macrophages, phagocytosing foreign bodies, and secreting cytokines, causing an inflammatory response and also initiating an adaptive immune response. The third line of defense is the acquired immune line of defense, including antigen capture and processing, antigen presentation, activation of B lymphocytes, and secretion of specific antibodies. At the same time, T lymphocytes are activated to kill abnormal cells. However, the target cells of ASFV are macrophages and DCs, and they encode a variety of proteins that inhibit the function of IFN. The inhibition of ASFV on the body’s immunity is reflected in various aspects, including the regulation of inflammatory response, the inhibition of antigen presentation, and the inhibition of apoptosis. Therefore, the development of ASF vaccine has a long way to go. It is necessary to fully understand the interaction between ASFV and the body’s immunity, and to find the key genes that can play a protective role. Currently, different vaccines have been tried against ASF. ASF LAVs can provide 100% protection against homologous strains, but there are still potential safety concerns. ASF vaccine needs more relevant basic research. In addition, subunit vaccines are also one of the current research priorities. In conclusion, screening ASF immunogenic proteins, identifying immune-related genes, and understanding the protective mechanism of ASF vaccines will facilitate the development of new ASF vaccines.

## Figures and Tables

**Table 1:** Summary of the most promising LAV candidates.

Genes	Strains	Genotype	Minimal Protective Dose	Route	Challenge	Gene Function	References
I177L	Georgia2007/1	II	10^2^HAD_50_	IM	Georgia2007/1	unknown	[19]
			10^6^HAD_50_	ON	Georgia2007/1		[20]
			10^2^HAD_50_	IM	TTKN/ASFV/DN/2019		[21]
A137R	Georgia2007/1	II	10^2^HAD_50_	IM	Georgia2007/1	unknown	[23]
I226R I226R	SY18	II	10^4^HAD_50_	IM	SY18	unknown	[22]
L7L-L11L	SY18	II	10^3^HAD_50_	IM	SY18	unknown	[18]
MGF505/360(6) ^1^ and EP402R	HLJ/18	II	10^3^HAD_50_	IM	HLJ/18	hemadsorbing and inhibition of type I interferon responses	[17]
			10^5^HAD_50_	ON			
EP402R	Ba71V	I	10^4^HAD_50_	IM	Ba71V	hemadsorbing	[86]
					E75		
					Georgia2007/1		

^1^: MGF505-1R, MGF505-2R, MGF505-3R, MGF360-12L, MGF360-13L, MGF360-14L.

## Data Availability

Not applicable.
